# Reusability of brilliant green dye contaminated wastewater using corncob biochar and *Brevibacillus parabrevis*: hybrid treatment and kinetic studies

**DOI:** 10.1080/21655979.2020.1788353

**Published:** 2020-07-07

**Authors:** Balendu Shekher Giri, Sudeshna Gun, Saurabh Pandey, Aparna Trivedi, Riti Thapar Kapoor, Rajendra Prasad Singh, Omar M. Abdeldayem, Eldon R. Rene, Sudeep Yadav, Preeti Chaturvedi, Neha Sharma, Ram Sharan Singh

**Affiliations:** a Department of Chemical Engineering and Technology, IIT(BHU), Varanasi, India; b Aquatic Toxicology Laboratory, Environmental Toxicology Group, Council of Scientific and Industrial Research-Indian Institute of Toxicology Research (CSIR-IITR), Lucknow, India; c Department of Chemical Engineering, NIT Durgapur, West, India; d Department of Chemical Engineering, Uiet CSJM University, Kanpur, India; e Amity Institute of Biotechnology, Amity University, Noida, India; f Department of Municipal Engineering, Southeast University, Nanjing, China; g Department of Water Supply, Sanitation and Environmental Engineering, IHE Delft Institute for Water Education, Delft, The Netherlands; h Department of Chemical Engineering, Bundelkhand Institute of Engineering & Technology (BIET), Jhanshi, India; i Amity Institute of Microbial Technology, Amity University, Noida, India

**Keywords:** Adsorption, biochar, brilliant green, corncob, wastewater

## Abstract

This work highlights the potential of corncob biochar (CCBC) and *Brevibacillus parabrevis* for the decolorization of brilliant green (BG) dye from synthetically prepared contaminated wastewater. The CCBC was characterized by proximate, Fourier-transform infrared spectroscopy, scanning electron microscopy, X-ray diffraction, and Brunauer–Emmett–Teller analysis, respectively. Different parameters affecting the adsorption process were evaluated. The experimental results were analyzed by the Langmuir and Freundlich isotherm models. Kinetic results were examined by different models; pseudo-second-order model has shown the best fit to the experimental data. Anew positive values of *ΔH^o^* (172.58 kJ/mol) and *ΔS^o^* (569.97 J/K/mol) in the temperature range of 303–318 *K* revealed that the adsorption process was spontaneous and endothermic. The present investigation showed that the bacteria immobilized with CCBC showed better BG dye degradation. The kinetic parameters, *μ_max_, Ks*, and μ*_max_,* were found to be 0.5 per day, 39.4 mg/day, and 0.012 L/mg/day using Monod model, respectively. The adsorbent with bacteria showed good potential for the removal of cationic BG dye and can be considered for the remediation of industrial effluent.

## Graphical abstract

**Figure:**
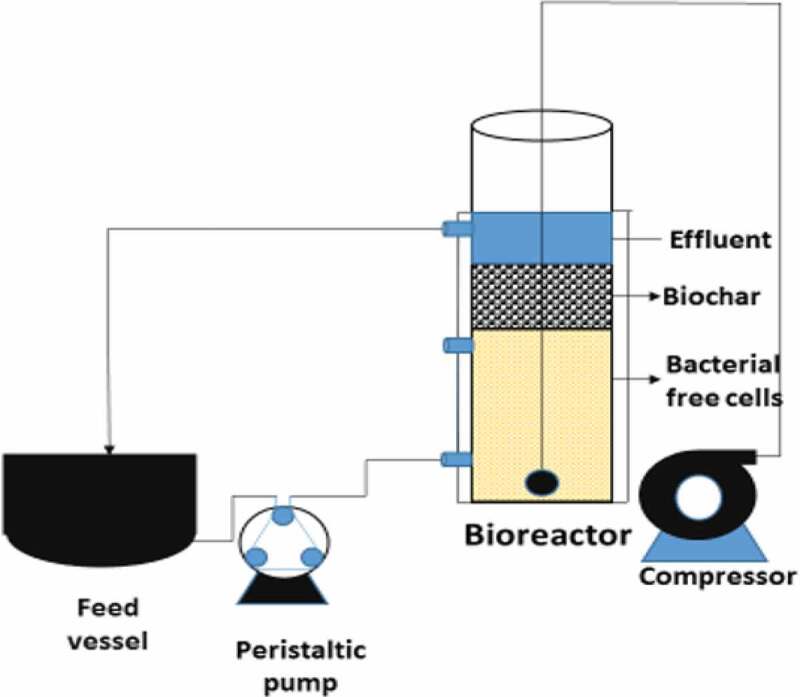

## 1. Introduction

Pollution due to rapid population growth and industrial development has largely contributed to the deterioration of water quality [1]. Textile industries are the main culprit of environmental deterioration. The textile sector consumes large volumes of water and generates wastewater that are usually contaminated with coloring material, salt, and other fixing agents [2,3]. The disposal of industrial effluents into water bodies, that is, without treatment or inadequate treatment, has shown to cause adverse impact on the environment and human health [4].The annual global production of dyes is 7 × 10^5^ tones [5,6], and in India, over 1.5 million liter of effluent containing dyes are released by the industries every day [7]. Azo dyes are one of the largest class of dyes with production estimated between 60 and 70% of all the dyeing materials produced annually [8]. Approximately 10–12% of the dyes are lost during the processing and disposed into industrial wastewater [9]. The availability of high-quality water is a prerequisite for good health, ecosystem, and sustainable economy [10]. In developing and developed countries, lack of accessibility to clean water is a topic of serious concern. The occurrence of dye in trace amounts (< 1 mg/l) is visible and undesirable in water which makes the water unfit for irrigation, domestic uses, and human consumption [11]. The colored water reduces sunlight penetration in water bodies, lowers its photosynthetic activities, causes eutrophication, oxygen deficiency, and disturbs the entire aquatic balance [12,13]. Dyes and their intermediate products adversely affect the health of human beings due to its carcinogenic, cytotoxic, mutagenic, and immune suppression effects [14].

Brilliant green (BG) cationic dye is widely used in the pulp and paper and dying industries [15]. The cationic dyes are more toxic when compared to the anionic dyes [15]. BG is utilized in manufacturing of ink and staining component for media. It is a highly toxic dye as its fatal dose is in the range of 50–500 mg/kg for human beings [16]. Additionally, studies showed that it causes adverse effect on rats by affecting their renal and reproductive systems [17]. Hence, BG has been linked to causing several health-related disorders, for example, skin and respiratory tract irritation, coughing, breath shortness, and irritation to the gastrointestinal tract, with symptoms of nausea, abdominal pain, and diarrhea.

Many physical–chemical methods such as ozonation [18], electro-flotation [19], electro-oxidation [20], nano-filtration membrane [21], reverse osmosis [22], coagulation-flocculation [23], ion-exchange [13], and adsorption [24,25] have been used for treating dye containing wastewaters. However, these technologies have some drawbacks: (i) complex operating procedures, (ii) intensive energy requirement, (iii) high operational cost, and (iv) production of secondary wastes [26–28]. Cost is an important parameter to select a proper adsorbent. Among the several techniques, adsorption using agricultural biomass has received considerable attention because of the following reasons: (i) agro-based wastes are available in large quantities at a cheaper price, (ii) demonstrate high dye removal rates, (iii) ability to treat concentrated dye containing effluent, and (iv) its reuse and recyclability [29–31]. In literature, different agro-wastes have been used for dye removal from effluents such as jute sticks [32], Jamun leaves [33], rice straw [34], wheat straw [35], banana peel [36], pine wood [37], etc. Maize (*Zea mays* L.; family: Poaceae), also known as corn, is a third largest food crop in India with an annual production of 24.3 million metric tons [38]. Huge quantities of corncobs are produced as agricultural waste during its processing [39]. Most of the corncob wastes are discarded or burnt and such non-engineering practices pollutes the environment [40,41].

The degradation of dyes using biocatalysts (ie microorganisms) is cost-effective and efficient wherein less sludge is produced; however, as the microbial processes are slow, its application is limited for the treatment of dye containing wastewater at the industrial scale [42]. Among the different microbes, bacteria exhibit fast and better degradation ability and they are capable of operating under harsh conditions. Datta et al. [43] reported 80% biodegradation of BG dye by *Micrococcus luteus* and *Pseudomonas syringae*. There is no previous research till date in the literature on the use of corncob biochar (CCBC) and *Brevibacillus parabrevis* for the removal of cationic dye BG. The operation of a continuous packed bed reactor is more efficient for real-time industrial effluent treatment [44].

The present study has been designed with an aim to determine the compatibility of using corncob and *Brevibacillus parabrevis* for removal of BG dye in batch and continuous packed bioreactors. The performance of the reactors was compared under free cell and immobilized cell condition and the reactors process parameters were optimized.

## 2. Materials and methods

### 2.1 Collection of corncobs and biochar preparation

Corncobs were collected from the local corncob vendor shop in Varanasi (after the grains were removed). The corncobs were initially washed several times with tap water and thrice with distilled water to remove contaminants from its surface. Then, they were cut into pieces and kept under sun light for 5–6 days to decrease moisture content. A pyrolysis reactor was used for biochar production from corncob. The nitrogen gas was purged in the reactor for maintenance of inert condition and it checks the ash production. The dehydrated corncobs were grinded and sieved to 210 μm particle size. Then, 2.0 kg of the corncob was pyrolyzed at 500*°C* for 4 h. Pyrolysis produces the solid carbon content known as biochar, liquid (bio-oil), and gaseous residue. The pyrolyzed biochar was treated with Milli-Q water and dried at 70°C for 2 h in an oven. The prepared biochar was kept in airtight plastic container.

### 2.2. Proximate analysis and characterization of biochar (FTIR, BET, SEM, and XRD analysis)

Proximate analysis of CCBC was evaluated to check its sturdiness toward thermochemical process. Then, 2 g biochar was placed in a crucible at 110°C in hot air oven for 1 h for calculation of moisture content. For volatile content, 2.0 g biochar was taken in a closed crucible and was kept in a muffle furnace at 900°C for 10 min. For the determination of ash content, 2.0 g of biochar was kept in an open crucible at 775°C for 1 h in the muffle furnace. Fixed carbon content was calculated according to the formula shown in Eq. (1):

Eq. 1
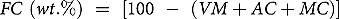

The presence of functional groups on CCBC before and after the adsorption of BG dye were determined by Fourier-transform infrared spectroscopy (FTIR) (NICOLET 5700 FT-IR, Tokyo, Japan) in wave number range of 400–4000 cm^−1^ using KBr pellet method.

Brunauer–Emmett–Teller (BET) (ASAP 2020 Micromeritics, USA) analysis was conducted to know surface area, pore size, and adsorption volume of CCBC. Morphology of CCBC surface was analyzed by scanning electron microscope (SEM) (QUANTA 200 F, The Netherlands).

X-ray diffraction (XRD) analysis was performed to identify the composition of minerals present and formed during the treatment using biochar by computer-controlled X-ray diffractometer (Philips Electronic Instruments, Tokyo, Japan) equipped with a stepping motor and graphite crystal monochromator. Crystalline minerals in the samples were identified by comparing diffraction data against a database compiled by the Joint Committee on Powder Diffraction and Standards [45].

### 2.3. Preparation of the Brilliant Green dye stock solution

BG was procured from Sigma-Aldrich Chemicals, India. BG, cationic dye, is derivative of triarylmethane dye (Table 1). The stock solution (1000 ppm) of BG dye was made initially and it was diluted to produce dye solution of required concentrations. The concentration of BG dye was measured at 626 nm using an UV-Vis double beam spectrophotometer (Elico SL 169, Hyderabad, India).

**Table 1.** Physico-chemical characteristics of Brilliant green (BG) dye used in the experiment including proximate and BET surface area analysis for corncob and corncob biochar.

Items	Values
Dyestuff	Ethyl green, Malachite Green G
Appearance	Dark green crystalline powder
IUPAC Name	[4-[[4-(diethylamino) phenyl]-phenyl methylidene] cyclohexa-2,5-dien-1-ylidene]-diethyl azanium; hydrogen sulfate
Empirical Formula	C_27_H_34_N_2_O_4_ S
Color Index Number	42,040
Molecular Weight	482.639 g/mol
Molecular Structure	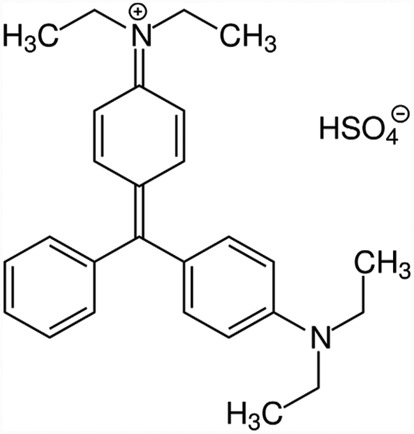
λ_max_ (nm)	626
**Proximate analysis for corncob biochar**	
Moisture content (*wt.%*)	1.2
Volatile matter (*wt.%*)	22.3
Ash content (*wt.%*)	5.8
Fixed carbon (*wt.%*)	70.7
**Surface characterization of corncob biochar**	BET Analysis
BET surface area (m^2^/g)	770.69
Pore volume (cm^3^/g)	0.406
Average pore diameter	2.873
**Thermodynamic parameters**	
Temperature (*K*)	303 (303 to 318)
Free Energy Change (∆*G^o^*) KJ/mol	−0.276 (−0.276 to −8.870)
*ΔH^o^* (kJ mol^−1^)	172.58
*ΔS^o^* (J/K mol)	569.97

### 2.4. Batch adsorption studies

#### 2.4.1 Batch adsorption experiment

In batch experiments, effects of different parameters including dye concentration (50–200 ppm), biochar dose (0.5–2.5 g), pH (5.0 to 9.0), and exposure time (30–210 min) were investigated at 120 rpm agitation speed for BG dye removal on produced CCBC. The 100 ml of BG dye solutions (50, 100, 150, and 200 ppm) were kept in four different Erlenmeyer flasks with different biochar amount (0.5, 1.0, 1.5, 2.0, and 2.5 g). Flasks were kept in incubator shaker at 120 rpm and OD was taken at λ_max_ = 626 nm for determining BG dye concentration in supernatant.

Eq. 2
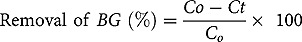

where, *C_o_* and *C_t_* are initial and final concentrations of BG dye at time *t* in mg/l.

#### 2.4.2. Point zero of charge

Point zero of charge was analyzed by the procedure of Rivera-Utrilla et al. [46]. The 100 ml of NaCl (0.01 M) solutions were prepared and their pH ranging from 2.0 to 12.0 was maintained by 0.10 M NaOH or HCl [16]. Then, 1.0 g of adsorbent was introduced into each solution and stirred at room temperature for 24 h before determining the pH_final_. The pH_PZC_ was calculated on the basis of curve *pH_final_ – pH_initial_ = f(pH_initial_).*

#### 2.4.3. Adsorption kinetics and isotherm studies

The kinetic parameters provide rate of adsorption, equilibrium time, and help in the modeling of adsorption processes [47]. Pseudo-first-order and pseudo-second-order models were used for the determination of kinetics involved in adsorption process. Pseudo-first-order reaction can be expressed as [48]:

Eq. 3
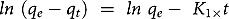

where *q_e_* and *q_t_* are BG dye amount adsorbed at equilibrium and time *t* and *k_1_* is pseudo-first-order adsorption rate constant (min ^1^). Pseudo-second-order reaction can be calculated by equation of Ho and McKay [49]:

Eq. 4
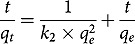

where *q_e_* is BG dye adsorbed on the adsorbent at equilibrium and *k_2_* is pseudo-second-order adsorption rate constant (g/mg/min).

Two isotherms were applied to evaluate adsorption equilibrium. BG dye (50 mg/l) 100 ml solution were taken with different adsorbent dosages to check the feasibility of the isotherm by comparing the adsorption capacity. Langmuir isotherm model exhibits that adsorption process occurs in a monolayer manner and energy of adsorption is uniform on the adsorbent surface at constant temperature [8]. Langmuir equation is given as:

Eq. 5
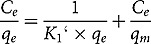

where *q_e_* (*mg/g*) = BG dye adsorbed at equilibrium, *q_m_* (*mg/g*) = amount of dye adsorbed, *C_e_* = dye concentration (mg/l) at equilibrium, and *K_1_ *= Langmuir constant.

Freundlich isotherm explains distribution of solute molecules between aqueous and solid phases at equilibrium. The Freundlich equation is expressed as:

Eq. 6
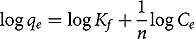

where, *K_f_* and *n* are Freundlich constants. Value of *n* shows nature of process if *n <* 1, chemical; *n* = 1, a linear; *n* > 1, a physical process.

#### 2.4.4. Determination of activation energy

Reaction rate depends on temperature and with increasing temperature molecular collision takes place. The relation between temperature and rate constant can be explained by equation:

Eq. 7
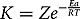

where *k* = rate constant, *Z* = proportionality constant, *E_a_* = activation energy for reaction, and *R* = gas constant.

The following equation is retrieved by using natural logarithm on both sides:

Eq 8
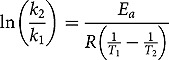

#### 2.4.5. Thermodynamic analysis

Changes in free energy, enthalpy, and entropy were determined for adsorption of BG on the CCBC.

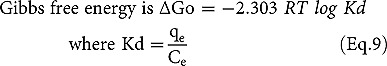

Eq. 10
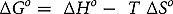

After rearranging the equation, *log K_d_ =*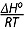-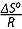. Using curve fitting method, the values of enthalpy (*∆H^o^*) and entropy *(∆S^o^*) were calculated.

### 2.5. Biodegradation of dye by bacterial cells by isolated bacteria

Bacterial species were isolated from the industrial effluent obtained from neighboring areas of the carpet industries in Bhadohi region, Uttar Pradesh, India. Nutrient agar medium was used as a growth medium. The solution (100 ml) was prepared by mixing 95 ml of nutrient agar; 5.0 ml of BG dye (50 mg/l) and 2.0 ml of isolated bacterial inoculum were also added and incubated at 30°C for 2 days. Diluted culture was spread on petri plate and bacterial colonies were observed after 48 h. These colonies were further sub-cultured six times to obtain pure strains. Two different bacterial isolates were labeled as B1 and B2. Control nutrient agar medium was also used without dye and bacterial culture. Two bacterial strains were assessed for BG degradation by by measuring the OD values using an UV-Vis spectrophotometer.

#### 2.5.1. Screening of best bacterial isolate and its biodegradation kinetics in free cell

Two bacterial samples were isolated from wastewater released from carpet industry and the isolate labeled as B2 showed better growth and degradation of BG dye during analysis with UV-Vis spectrophotometer at 626 (peak for testing BG dye) and 600 nm (to check bacterial growth). B2 was identified as *Brevibacillus parabrevis* by DNA sequencing technique.

The small amount of bacterial inoculum was added to different concentrations of BG dye for its degradation. The sample (3 ml) was centrifuged and filtered and its OD was taken by UV-Vis spectrophotometer at 626 nm. Decolorization (%) was calculated by using the following formula:

Eq. 11
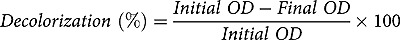

The kinetics of biodegradation was studied using the Monod’s model [50].

Eq. 12
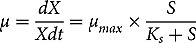

where *μ* and *μ_max_* are specific and maximum specific growth rate of bacteria; *K_s_* = half velocity constant (mg/l); *X* = bacteria concentration; *S* = limiting substrate concentration; *t* = time taken for degradation. The equation can be written a**s** follows [51]:

Eq. 13
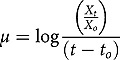

#### 2.5.2 Effect of inoculum size in free cell and packed bed bioreactor

The effect of inoculum on dye degradation was studied by varying it from 1.0 × 10^5^ to 5.0 × 10^5^ CFU/ml at 35 ± 2°C for 24 h for BG dye (50.0 mg/l). The 3.0 × 10^5^ CFU/mL was considered as an optimum inoculum size as dye degradation percentage was significantly increased at this inoculum size and degradation becomes constant beyond this range. A cylindrical borosilicate glass packed bed bioreactor (L = 48 cm and D = 8.3 cm) with flat bottom and proper grooving to hold the beds was designed. The feed was given from the top of the reactor through silicon tubes. CCBC was added between two grooves of the tube (6.0 cm) and was supported by metal sieves tied with muslin cloth above the packing material. Two outlet ports were provided, one below and the other above the packing for collection of the samples. The third outlet port at the base of the bioreactor serves the purpose of proper circulation of BG dye. The temperature of bioreactor was controlled by thermostat. The aerobic condition was maintained in the bioreactor by supplying decontaminated air through a diffuser placed at the bottom of the bioreactor.

## 3. Results and discussion

### 3.1. Proximate analysis and characterization of corncob biochar

Proximate analysis was conducted to analyze fixed carbon, volatile matter, moisture, and ash content in a CCBC. Proximate analysis data showed that adsorbent has 70.7% fixed carbon, 22.3% volatile matter, 1.2% moisture content, and 5.8% ash content (Table 1).

The functional groups present on CCBC surface were determined by FTIR spectroscopy. On comparing the FTIR spectra analysis of the corncob before and after BG dye adsorption, it was clear that the large number of functional groups present on the corncob surface facilitated BG dye adsorption (data not shown schematically). The broad peak with rounded tip at 3034 cm^−1^ in CCBC spectrum is assigned to O–H stretching of an alcoholic group of adsorbents. The peak at 2341 cm^−1^ might be due to O = C = O stretching of carbon dioxide. A band at 1257 cm^−1^ is allocated to C-O stretching of alkyl aryl ether group. At 3042 cm^−1^, peak corresponds to O–H stretching of alcoholic group. A clear peak at 1565 cm^−1^ indicates N-O stretching of nitro group and peak at 1176 cm^−1^ exhibits C–O stretching of tertiary alcohol group. Result also exhibits the presence of C-Cl and C-I stretching of halo compounds at wave numbers 846 and 455 cm^−1^, respectively (Figure S1a and S1b). The BET (Micromeritics, AAP 2020, surface area and porosity analyzer) analysis of CCBC was conducted. The result indicates that BET surface area is 770.69 m^2^/g, pore volume is 0.406 cm^3^/g, and adsorption average pore diameter is 2.873 nm (Table 1).

### 3.2. Surface studies using scanning electron microscopy and XRD analysis

**Figure 1.**
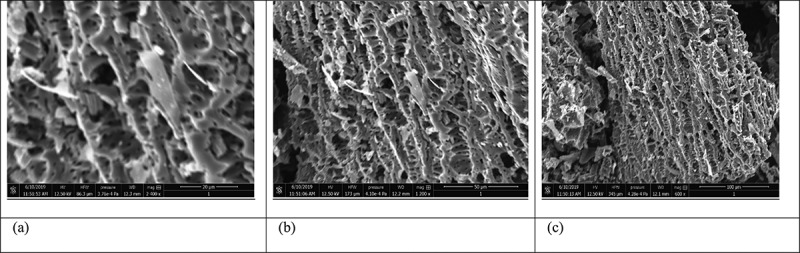
SEM images of corncob biochar (CCB): (a) before adsorption of Brilliant green dye on corncob biochar and (b) and (c) after adsorption of Brilliant green dye on corncob biochar.

**Figure 2.**
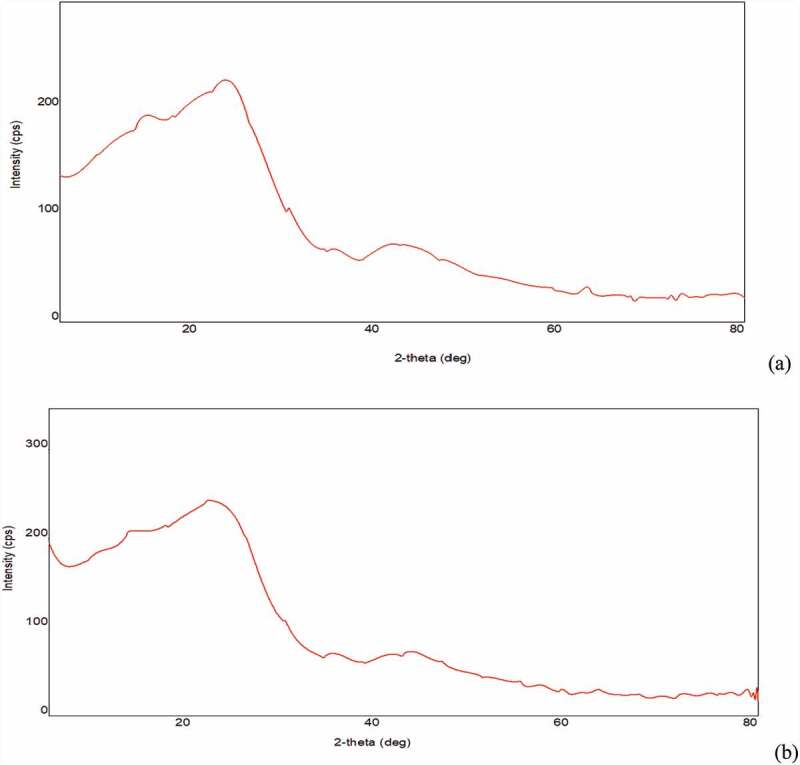
XRD images of corncob biochar (CCBC): (a) before adsorption of Brilliant green dye and (b) after adsorption of Brilliant green dye.

SEM technique makes it possible to visualize the morphology of the surface of the biochar. Micrographic images of CCBC obtained from SEM before and after adsorption BG dye adsorption have been displayed in Figure 1a and b, c, respectively. The corncob surface was very irregular, porous with cave type openings. The large number of pores were observed on the biochar surfaces, which promote BG adsorption [52]. SEM images clearly indicate that corncob can easily adsorb BG dye due to its adequate morphology. XRD measurements deal with the crystallinity of the sample. The X-ray diffraction pattern of CCBC indicates that biochar is amorphous in texture and contains silica (Figure 2 a and b). The presence of sharper peak was due to the presence of silica, whereas group of small peaks indicated the presence of cellulose [53].

### 3.3. Investigation of Brilliant green dye adsorption by corncob biochar

#### 3.3.1 Effect of pH

**Figure 3.**
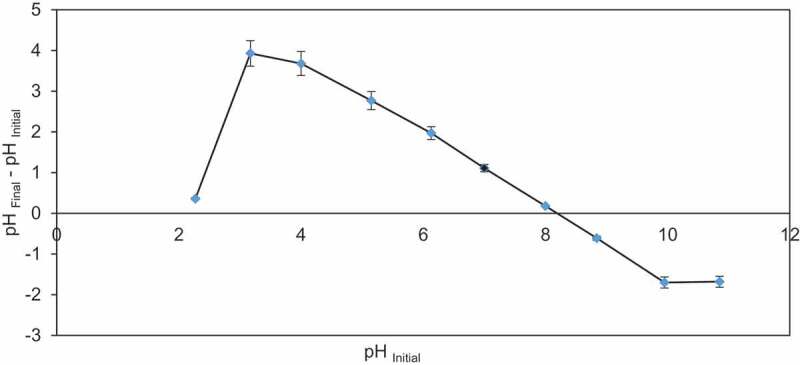
A graphical plot and calculation of the point zero of charge of corncob biochar (CCBC).

Impact of pH on BG was examined by altering pH from 5.0 to 9.0 at 27 ± 2°C with 50–200 mg/l BG dye solution. Maximum 99.9% removal of BG dye (50 mg/l) was observed by CCBC at high pH of 8.0, whereas 97.5, 99.4, and 99.4% dye removal were observed at pH 5.0, 6.0, and 7.0, respectively (Table 2). The pH controls the surface charge of the biochar and acts as indicator for adsorption of BG dye. Significant removal of BG dye was observed at high pH due to involvement of H^+^ ions in system. Increasing pH of the medium between 5.0 and 9.0 leads to a significant increase in the adsorption capacity of biochar (Table 2). Due to electrostatic force quick adsorption of BG dye by CCBC at high pH, negative charge increases on biochar surface with increase in pH and it causes deprotonation of the functional groups available on biochar. Deprotonated functional groups act as sites for binding of cationic dye. The pH_pzc_ of corncob adsorbent was 8.1 (Figure 3). The point of zero charge is related to pH value for which net electric charge of the surface of the material is neutral [45]. If the value of pH < pH_pzc_, it shows positively charged biochar surface and pH > pH_pzc_ indicates negatively charged surface [54]. Thus, pH_pzc_ value of 8.1 illustrates that CCBC can adsorb cationic dye if pH of the solution is more than point of zero charge.

**Table 2.** Effect of different parameters for brilliant green dye adsorption by corncob in batch tests.

Parameters and their values		BG dye removal efficiency (%)	q (mg/g)
pH (Condition: 50 mg/l, 1.0 g, 180 min)	5	99.9	4.99
	6	99.8	4.98
	7	99.4	4.96
	8	99.4	4.97
	9	97.5	4.88
Adsorbent dose (mg/l) (Condition: 50 mg/l, 180 min, pH = 5.0)	0.5	98.1	9.80
	1	98.1	4.90
	1.5	98.3	3.28
	2	98.8	1.98
Dye concentration (mg/l) (Condition: pH = 5.0, 1.0 g, 180 min)	2.5	98.8	2.47
	50	99.9	9.98
	100	99.9	19.97
	150	96.3	28.9
	200	21.9	8.77
Temperature (K) (Condition: 200 mg/l, 180 min, 1.0 g, pH = 5.0)	303	43.3	2.16
	308	45.4	2.27
	313	99.5	4.97
	318	99.2	4.96

#### 3.3.2 Effect of contact time and initial concentration of dye

**Figure 4.**
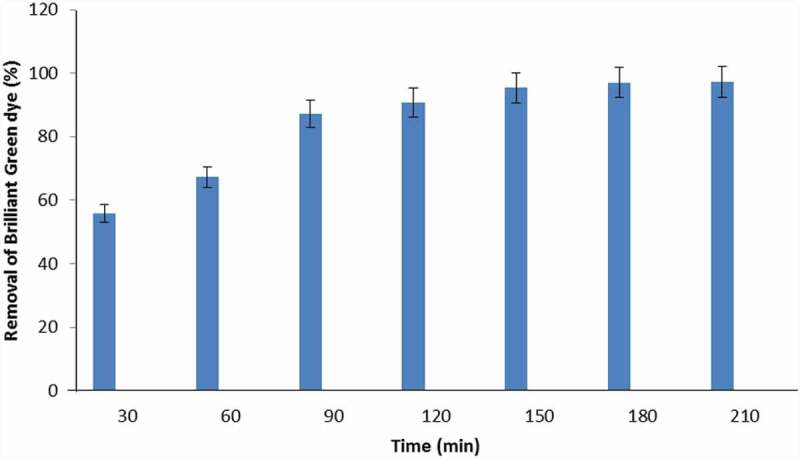
Effect of contact time on the removal of Brilliant green dye by corncob biochar (CCBC).

Effect of exposure time on BG adsorption was explored to know the equilibrium time of the process. As demonstrated in Figure 4, BG percentage removals 55.9, 67.2, 87.2, 90.7, and 95.4% were observed after 30, 60, 90, 120, and 150 min for 50 ppm dye concentration, respectively. Significant increase in BG dye removal rate was found by increasing adsorbent and adsorbate exposure time. Equilibrium time for adsorption process was found to be 180 min. A significant increase in adsorption capacity was reported at initial exposure period and it might occur due to the presence of vacant sites on the biochar. Mild increase in adsorption at later stage was due to the unavailability of sites [55]. Table 2 shows that adsorption capacity of biochar increases with dye concentration as it provides driving force and transfers BG dye to CCBC.

#### 3.3.3 Effect of adsorbent amount

Impact of CCBC amount on BG removal was examined by changing the biochar amount from 0.5 to 2.5 g. BG dye removal was increased from 98.1 to 98.8% when the biochar amount was increased from 0.5 to 2.5 g in 50 mg/l BG dye concentration as shown in Table 3. Maximum BG dye removal of 98.8% was observed with 2.0 g of CCBC. The surface area, structure of pores, and functional groups on biochar surface are the main factors that govern their abilities to adsorb dye. Increase in dye removal percentage with high adsorbent dose provides enhanced surface area with functional groups available for adsorption, which helps in easy piercing of dye on the adsorption sites [56]. However, adsorption capacity significantly reduced from 9.8 to 1.98 mg/g when biochar amount is increased from 0.5 to 2.5 mg/l. This illustrates aggregation of CCBC, which reduces surface area of adsorbent.

**Table 3.** Kinetic models (Pseudo-first-order and pseudo-second-order models) and adsorption isotherms (Langmuir and Freundlich) constants for the Brilliant green dye by corncob biochar.

Kinetic model	Equation	Constants	Values
Pseudo-first-order	ln (*q_e_-q_t_)* = ln *q_e_* – *k_1_ t*	*k_1_* (min^−1^)	0.0257
		*q_e_* (mg/g)	5.25
		*r^2^*	0.9737
Pseudo-second-order	*t*/*q_t_* = 1/*k_2_ q_e_^2^ *+ *t*/*q_e_*	*K_2_* (g/mg min)	0.005241
		*q_e_* (mg/g)	5.73
		*r^2^*	0.9945
Langmuir constants		*q_m_* (mg/g)	16.53
		*K* (L/mg)	0.52
		*R^2^*	0.9088
Freundlich constants		Adsorption intensity (*n*)	1.46
		Adsorption coefficient *K_f_* (mg/g)	2.04
		*R^2^*	0.9803
Monod model for growth Kinetics		*μ_max_* (per day)	0.492
		*K_s_* (mg/day)	39.4
		*µ_max_/K_s_* (mg/day)	0.012

#### 3.3.4 Effect of temperature

The adsorption of BG on CCBC was investigated at different temperatures such as 303, 308, 313, and 318 K after 180 min exposure period. BG dye (200 mg/l) showed 43.27 and 45.35% adsorption at 303 and 308 K, respectively. Additionally, BG dye adsorption was significantly increased to 99.17% at 318 K (Table 3). Temperature promotes adsorption process by enhancing diffusion rate of dye molecules in CCBC pores. By enhancing mobility of dye ions, adsorption capacity of adsorbent increases because retarding forces working on the diffusing ions decrease [57]. Increase in BG dye adsorption at high temperature was due to the positive value of *ΔH^o^* which suggests adsorption process was endothermic. Tavlieva et al. [58] also reported same results during adsorption of BG by white rice husk ash.

### 3.4. Adsorption isotherm and isotherm kinetics

**Figure 5.**
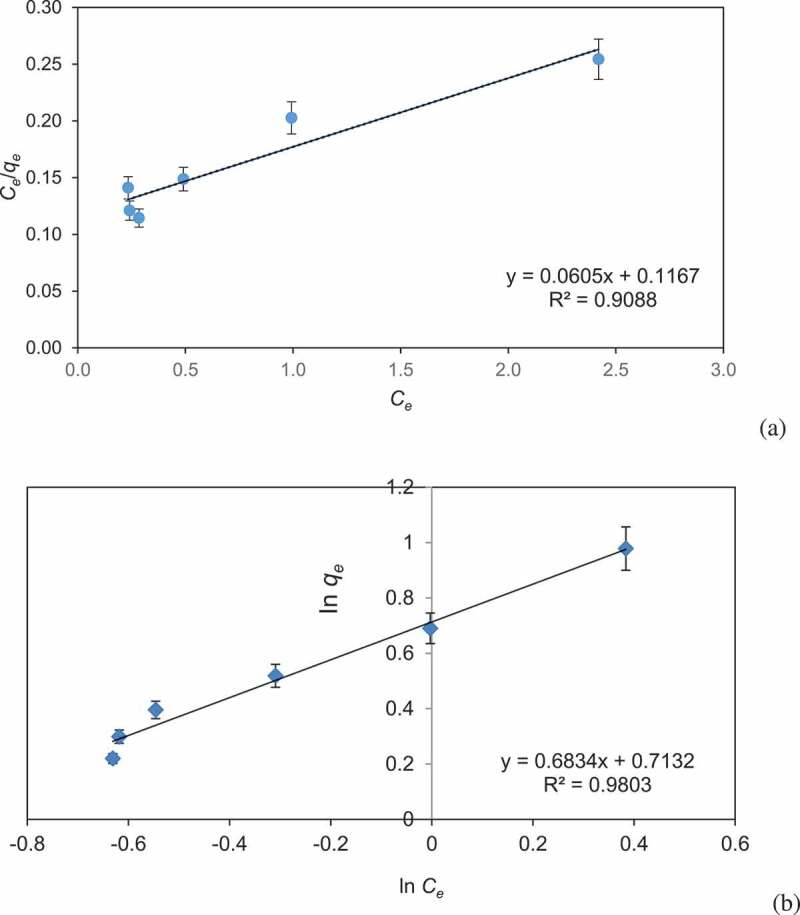
Adsorption isotherm of (a) Langmuir isotherm and (b) Freundlich isotherm for the adsorption of Brilliant green dye by corncob biochar (CCBC).

Adsorption isotherm model provides valuable facts on adsorption mechanism, adsorption capacity, and surface property of biochar. The isotherm modeling plays an important role in designing of effluent treatment system. Equilibrium results have been analyzed with Langmuir and Freundlich isotherm models (Figure 5a and b). In the present study, Freundlich model exhibited a better fitting model in comparison with Langmuir due to its higher correlation coefficient (*R^2^* = 0.9803). This exhibits multilayer coverage of BG dye on CCBC and the value of *n* was less than one, which shows physical nature of the adsorption process. Langmuir constants as calculated from equation showed the following values: *q_m_ *= 16.53 mg/g and *k* = 0.52 mg^−1^, (*R^2^ *= 0.9088) and Freundlich constants were *K_f_ *= 2.04 and *n* = 1.46 (*R^2^ *= 0.9803).

**Figure 6.**
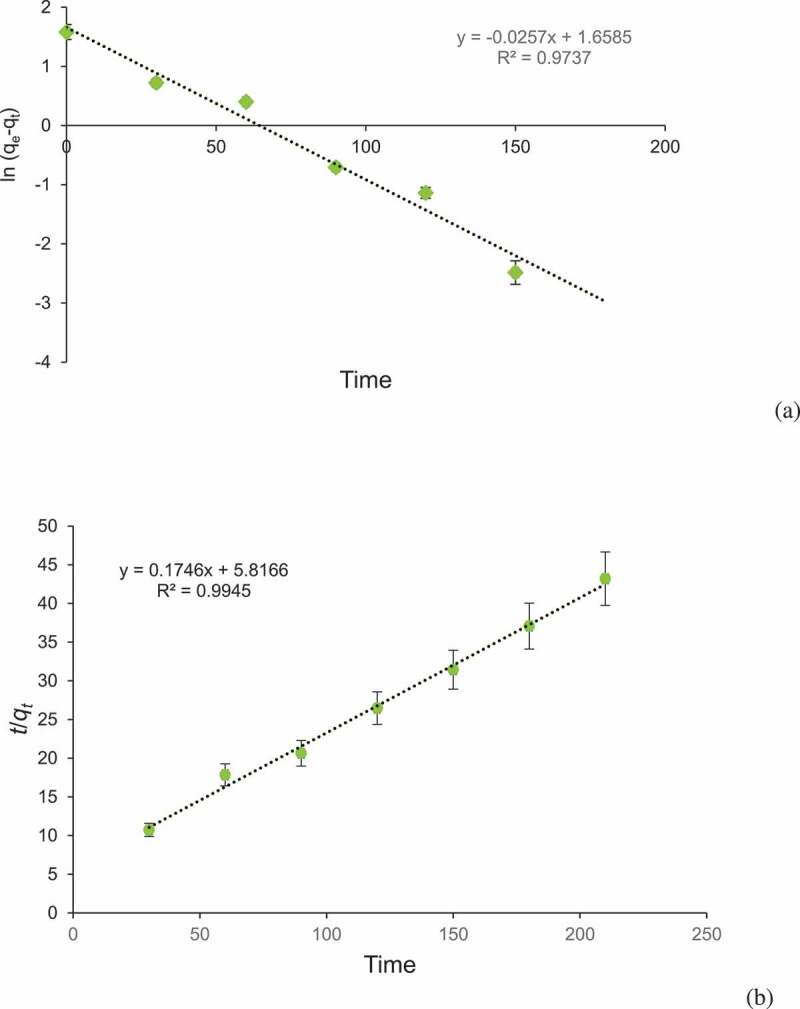
Adsorption kinetics of: (a) Pseudo first order reaction and (b) pseudo second order model fit for the adsorption of Brilliant green dye by corncob biochar (CCBC).

The kinetic studies provide knowledge regarding efficiency of adsorption process and reaction pathway. Pseudo-first-order and pseudo-second-order models were applied to examine adsorption of BG by corncob. The coefficient of determination (*r^2^*) was 0.9737 for pseudo-first-order and 0.9945 for pseudo-second-order model. Pseudo-second-order kinetic model was best obeyed due to its high correlation coefficient value (Figure 6a and b). The results showed that adsorption process was managed by sorption between dye molecules and corncob surface. The pseudo-second-order model was reported by different workers such as adsorption of crystal violet dye by wheat bran [59], tea dust [60], and *Tectona grandis* sawdust [61]. Adsorption of direct red dye on rice husks [62] and methylene blue on fruit shell of tamarind [63].

### 3.5. Activation energy and thermodynamic analysis of the adsorption process

**Figure 7.**
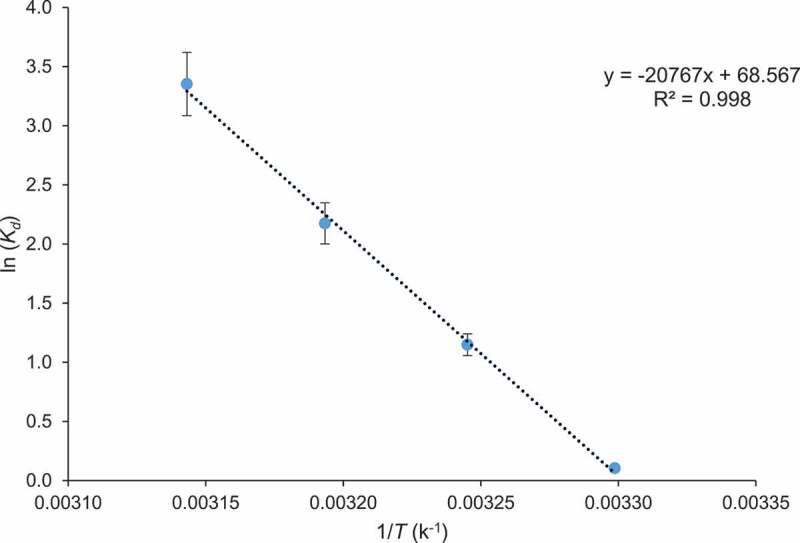
Thermodynamic parameters for the adsorption of Brilliant green dye by corncob biochar (CCBC).

Activation energy used in the adsorption process was evaluated by Arrhenius equation and value of *E_a_* was 588.46 kJ/mol, which indicates that CCBC possesses sites for binding of dye. The changes in Gibb’s free energy (*ΔG^o^*), enthalpy (*ΔH^o^*), and entropy (*ΔS^o^*) were analyzed to predict nature of adsorption. Experiments were conducted at different absolute temperatures such as 303, 308, 313, and 318 K. The results of thermodynamic studies are shown in Table 1 and Figure 7. The negative value of *ΔG^o^* at different temperature verified impetuous nature of adsorption of BG dye on CCBC. Furthermore, when the temperature increased from 303 to 318 K, the Gibbs free energy (*ΔG^o^)* decreased from 0.276 to 8.870 kJ mol^−1^, indicating that at high temperature adsorption process was impetuous [64]. The positive value of *ΔH^o^* (172.58 kJ/mol) verified endothermic nature of BG dye adsorption on biochar. The positive value of *ΔS^o^* (569.97 J/K mol) showed the affinity of the CCBC for dye and suggested fast adsorption process [65]. A conclusion can be drawn that BG dye adsorption onto CCBC was endothermic and spontaneous process which was consistent with the results observed in adsorption isotherm study.

### 3.6. Effect of concentration and time

**Figure 8.**
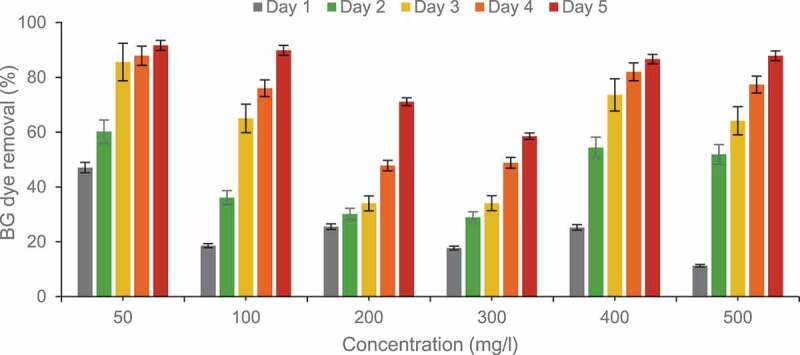
Effect of concentration and time on Brilliant green dye removal by free cells in batch experiments using corncob biochar.

Time directly affects the growth of bacteria and its application in dye degradation. Bacteria have a slower growth rate and require days for complete dye degradation. BG dye (50 mg/l) degradation rate was 47.1% after 24h. Degradation increased considerably to 85.6% after 72 h and maximum degradation 91.7% was observed in 5 days (Figure 8).

### 3.7. Kinetic studies with Monod model

Monod model was applied to study the kinetics of the bioprocess. A plot between *μ* and *S* showed a curvilinear relationship between specific growth rate and substrate concentration. The kinetic parameters, *μ_max_* and *K_s_,* were calculated from the graph and found to be 0.5/day and 39.4 mg/day, respectively. The low value of *μ_max_* and high value of *K_s_* showed a favorability of the reaction. *µ_max_/K_s_* shows the ability of degradation of bacteria in a bio-reactor [66]. The value of *µ_max_/K_s_* was 0.012 L/mg/day (Table 3). *Brevibacillus parabrevis* was able to degrade the high concentration of BG dye as its efficiency was observed by kinetic studies. The adsorption capacity of CCBC for BG dye adsorption was compared with the previous studies (Table 4). This indicates that corncob waste is cheap, promising, and valuable adsorbent for cationic dyes. Monod model was used to study the kinetic of the congo red dye using coconut shell biochar by plotting graph between *µ v/s S* and reported a curvilinear relationship between specific growth rate (*µ*) and substrate concentration. The kinetic parameters, *µ_max_* and *Ks,* were calculated from the graph and found to be 0.461/day and 39.44 mg/day, respectively [67]. The low value of *µ_max_* and high value of *Ks* showed a favoring ability of the reaction. Future studies in this research direction could also combine the treatment of wastewater contaminated with metals and different bioreactor configurations such as a bio-filter or a fluidized bed bioreactor should be tested to ascertain the performance in continuous systems [68–70].

**Table 4.** Literature reports on the adsorption capacity achieved by different biochar-based materials for the removal of Brilliant green dye.

Dye	Biochar material	Adsorption capacity (mg/g)	Reference
Brilliant Green	*Cempedak duria*	0.21	Dahri et al. (2015)
	*Psidium guajava* leave	1.08	Rehman et al. (2015)
	*Solanum tuberosum* peel	1.17	Rehman et al. (2015)
	Jack fruit peel	9.47	Nora et al. (2015)
	Spent tea leaves	9.57	Nora et al. (2015)
	Modified chitosan	10.9	Karaer and Uzun (2013)
	*Luffa cylindrica* sponge	18.0	Segun Esan et al. (2014)
	Corncob biochar (CCBC)	16.5	This study

## 4. Conclusions

The present work clearly shows the synergistic effect of corncob biochar and *Brevibacillus parabrevis* for removal of BG dye from textile industry effluent. Thermodynamic study indicated that the dye adsorption on CCBC was spontaneous and endothermic process. Results reflected that immobilized system was better than free cell system for the treatment of dye contaminated effluent. Hence, due to high efficiency and low treatment cost, this process can be recommended for effluent treatment at large scale.

## Highlights

- Brilliant green (BG) from wastewater was adsorbed using agro-waste corncob.
- Corncob biochar (CCBC) possessed a maximum surface area of 770.69 m^2^/g and a pore volume of 0.41 cm^3^/g.
- Maximum multi-layered adsorption capacity of BG on CCBC was 16.5 mg/g.
- Synergistic effect of CCBC and *Brevibacillus parabrevis* for dye removal was found to be favorable.
